# Biomarkers in acute myocardial infarction

**DOI:** 10.1186/1741-7015-8-34

**Published:** 2010-06-07

**Authors:** Daniel Chan, Leong L Ng

**Affiliations:** 1 Pharmacology and Therapeutics Group, Department of Cardiovascular Sciences, University of Leicester, Clinical Sciences Building, Leicester Royal Infirmary LE2 7LX, UK; 2 Leicester National Institute for Health Research Cardiovascular Biomedical Research Unit, UK

## Abstract

Myocardial infarction causes significant mortality and morbidity. Timely diagnosis allows clinicians to risk stratify their patients and select appropriate treatment. Biomarkers have been used to assist with timely diagnosis, while an increasing number of novel markers have been identified to predict outcome following an acute myocardial infarction or acute coronary syndrome. This may facilitate tailoring of appropriate therapy to high-risk patients. This review focuses on a variety of promising biomarkers which provide diagnostic and prognostic information.

Heart-type Fatty Acid Binding Protein and copeptin in combination with cardiac troponin help diagnose myocardial infarction or acute coronary syndrome in the early hours following symptoms. An elevated N-Terminal Pro-B-type Natriuretic Peptide has been well validated to predict death and heart failure following a myocardial infarction. Similarly other biomarkers such as Mid-regional pro-Atrial Natriuretic Peptide, ST2, C-Terminal pro-endothelin 1, Mid-regional pro-Adrenomedullin and copeptin all provide incremental information in predicting death and heart failure. Growth differentiation factor-15 and high-sensitivity C-reactive protein predict death following an acute coronary syndrome. Pregnancy associated plasma protein A levels following chest pain predicts risk of myocardial infarction and revascularisation. Some biomarkers such as myeloperoxidase and high-sensitivity C-reactive protein in an apparently healthy population predicts risk of coronary disease and allows clinicians to initiate early preventative treatment. In addition to biomarkers, various well-validated scoring systems based on clinical characteristics are available to help clinicians predict mortality risk, such as the Thrombolysis In Myocardial Infarction score and Global Registry of Acute Coronary Events score. A multimarker approach incorporating biomarkers and clinical scores will increase the prognostic accuracy. However, it is important to note that only troponin has been used to direct therapeutic intervention and none of the new prognostic biomarkers have been tested and proven to alter outcome of therapeutic intervention.

Novel biomarkers have improved prediction of outcome in acute myocardial infarction, but none have been demonstrated to alter the outcome of a particular therapy or management strategy. Randomised trials are urgently needed to address this translational gap before the use of novel biomarkers becomes common practice to facilitate tailored treatment following an acute coronary event.

## Introduction

Coronary artery disease (CAD) and its end result, myocardial infarction (MI) continue to be a significant cause of mortality and morbidity in the western world. Over the past 50 years, it has become clear that the cascade of thrombotic events following atherosclerotic plaque rupture causes occlusion of the coronary artery, interrupting blood supply and oxygen to myocardium thus resulting in infarction. Myocardial necrosis following infarction is followed by heart failure, myocardial rupture or arrhythmias. Early treatment of myocardial ischaemia to prevent necrosis with treatments such as fibrinolysis, coronary artery bypass grafting and percutaneous coronary intervention have improved outcome [1].

Over time it has become clear that in order for such treatments to be of maximal benefit, timely diagnosis is important. Here, biomarkers become important, to help us improve our diagnostic accuracy of the disease, as treatments are not without risk. Furthermore, biomarkers also provide prognostic information about the disease, which then aids clinicians in deciding how aggressively they need to treat the disease.

### Definitions

Biomarkers are measurable and quantifiable biological parameters which serve as indices for health and physiology assessments [2]. This includes disease risk and diagnosis. The diagnosis of acute myocardial infarction (AMI) [3] can be made with the detection of a rise/fall of cardiac troponin (at least one value above the 99^th ^percentile of the upper reference limit) and one of 1) symptoms of ischaemia, 2) electrocardiogram (ECG) changes of new ischaemia, 3) new pathological Q waves or 4) imaging evidence of new loss of viable myocardium.

Both the ECG and cardiac troponin are biomarkers, but the focus of this review will be on serum proteins/markers which have become increasingly important to improve our diagnosis of myocardial infarction, in some cases identifying people at risk of having an infarct and in others to predict long term prognosis following an actual event.

### What makes a good biomarker?

A good biomarker is something that is easily measured and can be used as a surrogate marker for disease and its severity [4]. For instance, blood sugar can be used to diagnose diabetes [5] whilst glycosylated haemoglobin (HbA1c) monitors blood sugar control. Because cardiovascular disease continues to be a huge burden in most countries, it is important to identify high risk patients in order to prevent morbidity or mortality in later life. Medications and treatments also come at a cost and therefore simple and cheap tests have become increasingly necessary to decide how to target treatment. A good biomarker will diagnose or predict risk accurately (that is, high specificity and sensitivity), promptly provide affordable but meaningful results, and should provide this incrementally over existing markers or clinical characteristics.

### Biomarkers in acute myocardial infarction

**Figure 1:**
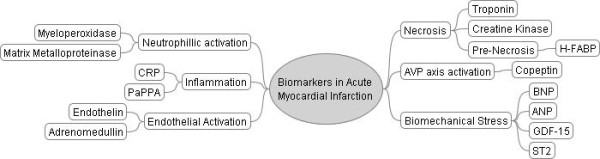
**Biomarkers associated with various pathophysiological processes associated with acute myocardial infarction**.

Some of these newer biomarkers and their relationship to various pathophysiological processes are depicted in Figure 1.

#### Diagnostic biomarkers

Two well known biomarkers in use for diagnosis of acute myocardial infarction are Creatine-Kinase-MB isoform and Cardiac Troponin. In 2000, Cardiac Troponin replaced CK-MB as the biomarker of choice for diagnosing a myocardial infarction [6]. Troponin is a protein released from myocytes when irreversible myocardial damage occurs. It is highly specific to cardiac tissue and accurately diagnoses myocardial infarction with a history of ischaemic pain or ECG changes reflecting ischaemia.

Cardiac troponin level is dependent on infarct size [7], thus giving clinicians an idea of the prognosis following an infarct. However, following reperfusion therapy, the actual troponin level can be misleading due to the *washout *phenomenon. Troponin levels peak at 12 hours, and stay elevated for 10 days or more. Whilst the use of Troponin for diagnosing AMI and risk stratification to aid decision making has revolutionised the management of patients presenting with chest pain, the 12-hour wait for the levels to peak remains the Achilles heel of this biomarker. Newer, more sensitive troponin assays [8] have been introduced to rectify this weakness. A positive Troponin is associated with increased risk of an adverse outcome at 30 days (HR 1.96, *P *= 0.003). In addition, the following two biomarkers may help facilitate early diagnosis of AMI, although neither has been compared with the newer high sensitivity troponin assays.

### C-terminal-provasopressin (Copeptin)

Copeptin is the more stable surrogate of arginine vasopressin (AVP), with well-known effects on osmoregulation and cardiovascular homeostasis [9]. Post AMI, vasopressin is thought to (1) increase peripheral vasoconstrictor activity thus increasing afterload and ventricular stress [10]; (2) increase protein synthesis in myocytes leading to hypertrophy [11] and (3) vasoconstriction of coronary arteries. These effects are mediated via the V1 receptor, whilst effects on the V2 receptor mediate water retention in the renal tubules. These receptors are now targets for pharmacological therapy [12,13]. Copeptin is released in stoichiometric proportion to vasopressin and is stable and easily assayed.

Copeptin can rule out MI earlier in addition to a negative Troponin T test [14]. At the time of presentation a copeptin level of < 14 pg/ml and a Trop T level of < 0.01 could rule out a myocardial infarction with an area under the curve (AUC) of receiver operating characteristic curve (ROC) of 0.97 (negative predictive value of 99.7%), thus obviating the need for monitoring and serial blood tests in a majority of patients. Copeptin is a good marker of neurohormonal stress, making it also useful in risk stratification in sepsis [15] and other diseases and hence is not specific to the cardiovascular system.

### Heart-Type Fatty Acid Binding Protein (H-FABP)

H-FABP is a low molecular weight protein involved in myocardial fatty-acid metabolism [16]. It is also found in small quantities in brain, kidney and skeletal tissue and levels can go up in acute ischaemic strokes and intense exercise. It is rapidly released early in myocardial infarction and necrosis into the cytosol. H-FABP has been shown in mouse studies to be an early marker of ischaemia [17] (before morphological evidence of myocardial necrosis) and can therefore help with diagnosis of MI earlier [17-19]. However, studies attempting to use H-FABP alone for early diagnosis of AMI have produced disappointing results. One review of six studies found that the pooled positive predictive value to be 65.8% and pooled negative predictive value to be 82.0% [20]. Other more recent studies demonstrated that H-FABP levels were clearly associated with the composite end point of death, myocardial infarction and heart failure at 10 months [21,22]. When levels of H-FABP were measured post-ACS and divided into quartiles, the top quartile was associated with all-cause mortality 6.59 times higher than the lowest quartile, after adjusting for hsCRP and Troponin. In fact, when added to Troponin for risk stratification, a negative troponin and H-FABP level < 5.8 mcg/L was associated with zero mortality at six months; a negative Troponin but H-FABP level > 5.8 mcg/L was associated with a 4.93-fold increase in risk of death and 7.93-fold increase in risk if Troponin was positive and H-FABP > 5.8 mcg/L.

#### Prognostic biomarkers

Before broaching the subject of biomarkers it is important to note that as a result of various randomized control trials and registry studies, various risk factors have been identified and entered into scoring systems that allow a clinician to risk stratify disease [23]. Popular tools include the TIMI score [24], derived from the Thrombolysis in Myocardial Infarction study, and the PURSUIT score [25] (from Platelet glycoprotein IIb/IIIa in unstable angina: Receptor sUppression using Integrillin Therapy). The GRACE score is another particularly robust clinical tool [26], which uses clinical indicators to calculate risk, (from the Global Registry of Acute Coronary Events study), utilizing weighted information about renal dysfunction, haemodynamic status, age, Killip Class, cardiovascular history, and history of a cardiac arrest, as well as elevated cardiac enzymes and type of ECG changes. On its own this score has an excellent c-statistic of 0.84 for predicting in-hospital death.

Newly introduced biomarkers should complement and have incremental prognostic value over and above these simple risk scores. It is therefore no surprise that biomarkers providing prognostic information following an acute coronary syndrome reflect the various physiological pathways described in the GRACE score (for example, haemodynamic status vs. biomechanical stress and neurohumoral pathways). Currently, the only accepted biomarker affecting a change in management of a patient with an acute coronary syndrome is the cardiac troponin.

### 1) Biomarkers of biomechanical stress

#### BNP/NTproBNP

One of the best known biomarkers of biomechanical stress is the B-type Natriuretic Peptide (BNP). Secreted by the ventricles in response to cardiomyocytes under tension [27], BNP binds and activates receptors causing reduction in systemic vascular resistance, central venous pressure and natriuresis. BNP has been studied extensively and provides prognostic information following an MI [28-30]. This biomarker has a short half-life but is released with the N-terminal portion of the pro-BNP peptide (NTproBNP), a peptide much more stable in serum and can be measured easily [31]. The understanding of its biochemistry is far from complete, in particular post-translational metabolism of the peptides, which may affect accurate determination of the levels of active BNP [32].

NTproBNP/BNP provides incremental information on cardiovascular death at one year in the older population above and beyond GRACE score [33]. On its own, it is at least as good as the GRACE score when predicting in-hospital mortality following AMI [34]; it also improves the accuracy of the prognosis when added to the GRACE score. In Non ST-elevation acute coronary syndromes (NSTEACS), this biomarker predicts in-hospital and 180 day death or heart failure [35]. Studies are summarised in Table 1[28-30,34-39].

**Table 1:** Summary of studies using BNP or NTproBNP for ris stratification of AMI

Study Pop	N	Endpoints	Thresholds	Odds ratio or Hazard Ratio	Ref
ACS-TIMI16	2,525	Death (30 day, 10 months) HF(10 months) MI (10 months)	Quartiles BNP > 80 pg/ml	1, 3.8, 4.0, 5.8 Approximately 2.7 Approximately 2	[28]

AMI	70	Death (18 months)	Median (>59 pg/ml)	Approximately 2.5	[29]

AMI-CONSENSUS	131	Death (one year)	75^th ^centile BNP 33.3 pmol/L	Approximately 1.36	[30]

ACS	609	Death	Median	2.4	[37]

NSTEACS	1,483	Death (in hosp) (180 days)	BNP > 586 pg/ml	(1.7) (1.67)	[38]

FRISC-II	2,019	Death	Top Tertile	4.1(invasive) vs 3.5(non-invasive)	[36]

TACTICS-TIMI18	1,676	Death (six months) HF (30 days)	BNP > 80 pg/ml	OR 3.3 OR 3.9	[35]

ACS	1,033	Death (30 day) (six months)	Quartiles	(2.24) (1.84)	[34]

AMI	473	Death	Median	OR 3.82	[39]

The TACTICS-TIMI 18 study [35] randomized 1,676 patients to conservative vs. early invasive therapy. Patients' BNP was measured within 24 hours and compared. This study found that the six-month mortality if BNP was below versus above cut-off of 80 pg/ml was 1.4% versus 8.4%, and risk of mortality or congestive heart failure below versus above cut-off was 3.6% vs. 16.3%. However, like another study [36], it did not identify patients who would benefit from early invasive revascularisation.

#### Mid-Regional pro-Atrial Natriuretic Peptide (MRproANP)

Like BNP, ANP has similar neurohormonal effects and has a similar secretory profile post AMI. Prior studies have attempted to accurately measure levels of ANP and N-ANP, with limited success [30,40]. N-ANP has been demonstrated to be associated with late mortality following AMI [41]. Such early N-ANP assays were often affected by interferences and instability of analyte. Because of disappointing results, ANP was thought to provide limited prognostic information. However, the discovery of the novel MRproANP fragment [42], a substantially more stable peptide compared to N-ANP and ANP [43] due to the assay epitopes being located internally on the proANP molecule (and hence stability to exoprotease activity), has led to the finding that MRproANP is at least as good at predicting death and heart failure as NTproBNP [44]. When MRproANP levels were divided into quartiles, the top quartile was associated with a hazard ratio (HR) of 3.87 (vs. NTproBNP HR 3.25) predicting death at follow-up. Both biomarkers had similar AUC of ROC (0.83). MRproANP is emerging therefore to be an important predictor of adverse events following an AMI.

#### Growth Differentiation Factor-15(GDF-15)

GDF-15 is a member of the Transforming Growth Factor Beta cytokine superfamily. It is not normally expressed in the heart, but under episodes of stress (for example, ischaemia and reperfusion) its levels go up in a variety of tissues, including cardiomyocytes. It has an antihypertrophic effect, demonstrated in knockout mice which develop early cardiac hypertrophic growth following pressure overload [45]. GDF-15 provides prognostic information following an MI or ACS. One study found that increasing tertiles of GDF-15 levels in patients presenting with NSTEACS was associated with an increasing risk (1.5%, 5% and 14.1% respectively) of death at one year (AUC of ROC 0.757) [46]. Studies are summarised in Table 2[46-49].

**Table 2:** Summary of studies using GDF-15 for risk stratification of AMI

Study Pop	N	Endpoints	Thresholds	Odds ratio or Hazard Ratio	Ref
GUSTOIV (NSTEACS)	2,081 + 429	Death (one year)	Tertiles < 1,200 ng/L, 1,200 to 1,800 ng/L > 1,800 ng/L	1.5%, 5%, 14.1%	[46]

FRISC-II (invasive vs conservative)	2,079	Death or MI (2 yrs)	Invasive at > 1,800 ng/L Invasive 1,200 to 1,800 ng/L Invasive < 1,200 or Trop -ve	HR 0.49 (risk reduction) HR 0.68 No benefit	[47]

ASSENT-2/plus (STEMI)	741	Death (1 yr)	Tertiles < 1,200, 1,200 to 1,800, > 1,800	2.1%, 5.0%, 14%	[48]

AMI	1,142	Death or HF (1.5 yr)	<1,470 ng/L, >1,470 ng/L	HR 1.77	[49]

These findings were validated in ST-elevation MI (STEMIs) [48] and prospectively validated in an unselected group of patients with AMI, where GDF-15 was found to be independently predictive of adverse events (death and heart failure) [49]. One study (FRISC-II) which randomized patients to conservative and early invasive strategy in patients with NSTEMI found GDF-15 to predict death or recurrent MI in the conservative group but not in the invasive group [47] suggesting that GDF-15 improves patients selection for early invasive strategy. It also directly compared the use of Troponin T vs the use of GDF-15 to select patients for early invasive therapy. Troponin-positive patients but with a GDF-15 level < 1,200 ng/L had no mortality benefit from early invasive therapy.

However, GDF-15 is not specific for cardiovascular disorders and has been found to be elevated in a variety of malignancies (prostate, colon, glial).

#### ST2

ST2 is an IL1-receptor-like protein which was found to be elevated in serum of hearts under mechanical stress [50]. ST2 predicts cardiovascular death following ACS [51]. ST2 turned out to be the target for an Interleukin called IL-33 which seems to have a cardioprotective role, and only appears when myocytes are under biomechanical stress [52]. In mouse studies, IL-33 was found to markedly antagonize angiotensin-II and phenylephrine-induced cardiomyocyte hypertrophy. It is thought that ST2/IL33 interaction also reduces atheroma burden [53]. Post AMI though, it correlates somewhat with NTproBNP [54], and both these biomarkers predict death after MI (at six months) or heart failure. Investigations into the use of IL33/ST2 pathway activation as a therapeutic target are still ongoing [55]. ST2 is also elevated in acute asthma [56] and autoimmune disease [57]. The specificity of ST2 to myocardial tissue stretch will need to be determined before it can be used at the bedside [58].

#### ET1/CTproET1

Endothelin-1 or the more stable C-Terminal portion of pro-Endothelin-1(CTproET1) has also been found to be predictive of death or heart failure following an AMI [59]. ET1 is a potent vasoconstrictor peptide found originally in vascular endothelial cells but has subsequently been isolated in pulmonary, renal and smooth muscle cells [60]. It activates ETA and ETB receptors; ETA receptors are located predominantly on smooth muscle tissue of blood vessels, mediating vasoconstriction and sodium retention, whereas ETB receptors are located predominantly on endothelial cells mediating nitric oxide release, natriuresis and diuresis [61]. Endothelin appears to be detrimental post-MI, extending the infarct [62] and reducing coronary blood flow [63]. It is also grossly elevated following cardiogenic shock [64]. ET-1 is very unstable and measuring its levels can be problematic due to binding with receptors and other proteins. However CTproET1 is a stable by-product of the release of the precursor which indirectly measures activity of the endothelial system. ET1 is increased in proportion to the severity of the disease post AMI [65,66]. Likewise CTproET1 is also elevated post-MI, and levels above the median predict death or heart failure (HR 5.71, *P= *0.002). This variable is independent of age, Killip class and past medical history. Plasma concentration of CTproET-1 peaks at Day 2 [59].

### 2) Biomarkers of neurohormonal pathway activation

#### Mid-Regional-pro-Adrenomedullin (MRproADM)

Adrenomedullin was first identified in human phaeochromocytoma cells [67]. It is highly expressed in endothelial cells [68]. Adrenomedullin mediates an increase in cAMP with resultant vasodilatation and hypotension [69]. Its other roles have not been well defined, but some have suggested a cardioprotective role at the time of the insult. The activity of adrenomedullin in the cardiovascular system is similar to that of BNP; that is, increase of nitric oxide production causing vasodilatation, natriuresis and diuresis [70-72]. Like BNP it is released in proportion to the severity of heart failure [73,74], and is inversely related to the left ventricular ejection fraction (LVEF)[75,76]. Adrenomedullin (ADM) is difficult to measure in plasma as it is partially complexed with complement [77]; in addition it is also rapidly cleared from the circulation. Indirect quantification of this peptide can be made by measuring the mid-regional fragment of the proAdrenomedullin peptide, which is more stable and secreted in equimolar concentrations as ADM. Initial studies with ADM have produced conflicting results as to the prognostic value of the peptide [40]. However, a recent study using the more stable MRproADM has shown that post AMI, increased MRproADM was associated with death, heart failure or both at one year, over and above information gained from NTproBNP alone [78]. Combining the two markers increased the AUC of the ROC from 0.77 and 0.79 to 0.84. MRproADM is very similar to NTproBNP, it is higher in females than males, and is increased with age.

#### Copeptin

The same study group also investigated Copeptin as a prognostic biomarker. They found that Copeptin predicted mortality or heart failure at 60 days post AMI [79]. In addition, the relationship of copeptin to LV dysfunction persisted for a prolonged period after the acute event [80]. Copeptin provided complementary prognostic information to NTproBNP, increasing the AUC of the ROC from 0.76 and 0.79 to 0.84.

**Figure 2:**
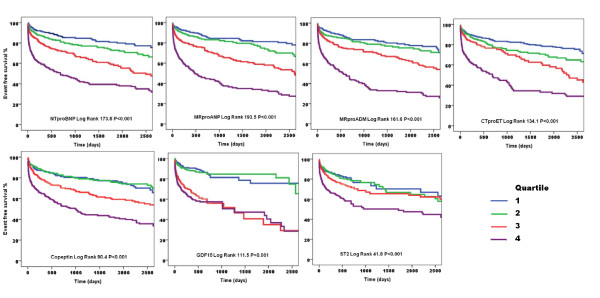
**Kaplan-Meier survival curves for quartiles of various biomarkers in 1,455 unselected AMI patients**.

Figure 1 is a summary of the Kaplan-Meier event free survival curves for some of the above mentioned biomarkers (namely NTproBNP, MRproANP, MRproADM, CTproET, copeptin, GDF-15 and ST2) in a prospectively collected cohort of AMI patients (derived from the Leicester Acute Myocardial Infarction Peptide (LAMP) study, illustrating the events associated with biomarker quartiles over about seven years. All of these biomarkers significantly predict major adverse events following AMI (Figure 2).

### 3) Biomarkers of plaque instability and inflammation

#### HsCRP (High-sensitivity C-reactive Protein)

Acute coronary syndromes are caused by *vulnerable plaques*. It is thought that one of the driving forces causing atheromatous plaques to rupture or erode, causing a cascade of events leading to coronary artery occlusion, is inflammation in the plaques. An elevated C-reactive protein measured in seemingly healthy adults was associated with increased cardiovascular risk [81,82]. CRP itself mediates atherothrombosis [83-87]. This is supported by a fairly large body of evidence. Newer, higher sensitivity assays of CRP that detect lower levels of CRP (<5 mg/L) risk stratify patients into low, intermediate and high risk, with intermediate and high risk individuals benefiting from aggressive therapy [88]. While the benefits of HsCRP testing in a primary setting to screen for ischaemic heart disease is very clear, its use post-ACS or -MI is less clear. CRP is elevated post-acute coronary syndrome almost exclusively in the setting of myocardial necrosis indicating the level of myocardial inflammation.

One study found that CRP measurements (taken between 12 and 24 hours post event) predicted occurrence of heart failure (HR = 2.6, *P *= 0.04) and death (HR = 2.7, *P *= 0.02) post-MI [89]. Elevated peak CRP in the early phase of MI was related to early mechanical complications, including cardiac rupture [90], ventricular aneurysm and thrombus formation. CRP levels post-MI peak at two to four days, then take 8 to 12 weeks to subside to baseline levels. Interestingly, CRP levels post acute MI do not predict re-infarction. Additional acute coronary events can only be predicted after CRP levels have receded to baseline levels (after about 12 weeks).

One of the difficulties with CRP is that it is non-specific in the presence of other inflammatory conditions (rheumatoid arthritis, malignancy, vasculitis). A new assay for Human Pentraxin 3 is now available. Human Pentraxin 3 is an isoform which is secreted exclusively in vascular endothelium and therefore may be more specific to the vascular plaque inflammatory activity [91]. It remains to be seen if this biomarker can provide incremental information.

#### Myeloperoxidase (MPO)

Leucocytes play a central role in atherosclerotic plaque rupture [92-96]. Myeloperoxidase in leucocytes may activate metalloproteinases and inactivate plasminogen activator inhibitor. Leucocytes also consume nitric oxide catalytically, causing vasoconstriction and endothelial dysfunction. Myeloperoxidase has been found in atheromatous plaques [96]. Patients with chronic angina have circulating neutrophils with large quantities of MPO, which decrease substantially post-ACS [97].

One trial showed that post-acute coronary syndrome MPO levels higher than median predicted future death and MI at one year [98]. It also found that after an AMI, MPO levels peak early, then decrease over time and do not correlate with Troponin or the neutrophil count. It is not affected by fibrinolytic therapy but it is unclear if it is affected by Primary Intervention. MPO levels do not predict heart failure. MPO levels higher than the median, though, predict death or MI after one year [99,100], whether or not NTproBNP level is below or above median. The risk is much higher if both MPO and NTproBNP levels are above the median.

Two population studies show that MPO (and CRP) in healthy individuals are both associated with future development of CAD [101,102].

Collectively the current evidence supports the need for further studies into the actual role of MPO, and whether elevated MPO levels in the serum directly correlates with MPO released from circulating neutrophils.

#### Pregnancy associated Plasma Protein A (PaPPA)

PaPPA is a proatherosclerotic metalloproteinase [103] which is highly expressed in unstable plaques and their extracellular matrices [104]. It is not expressed in stable plaques. Circulating PaPPA has been found to be much higher in unstable angina and AMI, correlating also with insulin-like growth factor and CRP, but not with Troponin. Interestingly, PaPPA > 2.9 mIU/L predicts a 4.6-fold increase in risk of cardiovascular death, MI or revascularisation even without a raised Troponin [105]. Its mode of action of cleaving insulin-like growth factor 1 (IGF-1) was found to counteract endothelial dysfunction by binding to high affinity binding sites in the endothelium which then triggers nitric oxide release. PaPPA has been isolated in other damaged tissue promoting repair, giving it an inflammation repressor role [106].

Like CRP, PaPPA is expressed when there is a heavy burden of unstable atheromatous plaque, including in carotid arteries [107]. It also predicts risk of cardiovascular death [105]. Unlike CRP, it does not predict heart failure. Instead, it predicts future MI and revascularisation. Evidence for the use of this biomarker clinically remains scarce and whilst promising, more studies and standardized assays will be needed to improve its clinical utility.

### 4) Other Novel biomarkers

#### MMP9, MMP2, TIMP1

The structural integrity of myocardial Extracellular Matrix (ECM) is dependent on endogenous zinc-dependent endopeptidases known as matrix metalloproteinases (MMP). These enzymes are regulated by tissue inhibitors of metalloproteinases (TIMPs). MMPs may degrade myocardial ECM leading to the development of LV dilatation and heart failure and their inhibition in experimental models of AMI has been associated with reduced LV dilatation and wall stress. Although NTproBNP, TIMP1 and MMP 9 were associated with cardiovascular death, heart failure or both, they were not associated with re-infarction [108]. MMP2 is also elevated post MI [109] and is associated with poor prognosis [110]. MMP3 peaks at 72 hours and plateau levels are associated with increase in LV volume and a lower ejection fraction at follow up [111].

#### Stability of biomarkers

Many of the new biomarkers introduced have enhanced stability *in vitro*, so that preanalytical contributions to variation are minimised to some extent. For example, the midregional epitopes assayed in MRproANP and MRproADM are less susceptible to exoprotease action, and prohormone fragment assays tend to be more stable than assays for the actual hormone (for example, NTproBNP, CTproET1, copeptin).

## Future directions

Although there are large numbers of emerging novel biomarkers, our understanding of the roles and biochemistry of these various peptides in the disease process is still fairly limited. It is difficult to draw specific conclusions from the current body of evidence regarding the mechanisms through which a biomarker could affect the prognosis. Many of the studies use death or major adverse cardiovascular events as endpoints because they are easy to measure, but either of these endpoints could be a culmination of a variety of pathophysiological processes. As such, currently available biomarkers have not been able to add much to helping us tailor our treatment (over and above Troponin). Randomised trials based on the use of biomarkers to alter therapy would be very informative. Although there is evidence that combining biomarkers may increase the accuracy of the tests, the best combinations for diagnosis or prognosis need to be defined. Some analogies can be drawn from heart failure studies; NTproBNP has been used as a biomarker for diagnosis of heart failure [112]. It does not provide clinicians with information about the aetiology nor which specific treatments to initiate. It does however guide therapy [113], as a surrogate marker of disease severity.

There is some evidence that a multi-marker strategy can improve diagnosis, risk stratification and prognostication of patients [114]. Of the above biomarkers, the ones most likely to be adopted into bedside practice in the near future are NTproBNP, MRproANP,MRproADM, copeptin and GDF-15. The theoretical benefit of a multi-marker approach would be tailored therapy as each biomarker would measure a separate disease sub-process. A multi-marker panel of tests could then be used to create an algorithm to aid clinical-decision making. We are, however, a long way from this eventual goal.

## Abbreviations

ADM: Adrenomedullin; AMI: Acute Myocardial Infarction; ANP: Atrial natriuretic peptide; AVP: Arginine vasopressin; BNP: B-type natriuretic peptide; CAD: Coronary artery disease; CTproET1: C-terminal pro-endothelin 1; CKMB: Creatine kinase MB isoform; ECG: Electrocardiogram; ECM: Extracellular matrix; ET1: Endothelin-1; ETA: Endothelin receptor type A; ETB: Endothelin receptor type B; GDF-15: Growth differentiation factor 15; GRACE: Global registry of acute coronary events study; H-FABP: Heart type fatty acid binding protein; HsCRP: High sensitivity C-reactive protein; IGF-1: Insulin-like growth factor 1; IL-33: Interleukin 33; LV: Left ventricle; LVEF: Left ventricular ejection fraction; MI: Myocardial infarction; MMP: Matrix metalloproteinase; MPO: Myeloperoxidase; MRproANP: Mid regional pro-atrial natriuretic peptide; MRproADM: Mid regional proadrenomedullin; N-ANP: N-terminal pro-atrial natriuretic peptide; NSTEACS: Non-ST elevation acute coronary syndromes; NTproBNP: N-terminal pro-B type natriuretic peptide; PaPPA: Pregnancy associated Plasma Protein A; ROC AUC: Area under the receiver operating characteristic curve; TIMI: Thrombolysis in Myocardial infarction study; TIMP: Tissue inhibitors of metalloproteinases; Trop: Troponin

## Competing interests

Prof. Leong Ng has submitted patents on behalf of the University of Leicester on some of the biomarkers in this review, and has acted as a consultant to and received grants in aid from BRAHMS AG and Unipath PLC in the past. Dr. Daniel Chan has no competing interests to declare.

## Authors' contributions

LN recruited the patients in the reported studies, performed the analyses and drafted the manuscript. DC participated in the analyses and drafting of the manuscript and figures.

## Pre-publication history

The pre-publication history for this paper can be accessed here:

http://www.biomedcentral.com/1741-7015/8/34/prepub
